# Supplementary material for: Subcritical Transmission in the Early Stage of COVID-19 in Korea
Source: Int J Environ Res Public Health. 2021 Jan 31;18(3):1265. doi: 10.3390/ijerph18031265 (PMC7908312; doi:10.3390/ijerph18031265)
Supplement: Supplementary file 1 [file ijerph-18-01265-s001.pdf]

International Journal of
Environmental Research and Public Health

MDPI

Table S1. COVID-19 Patient information in Republic of Korea.

ID	Source of infection	Sex	Travel History	Symptom onset	Confirmation	Discharge	Exposure	Entry to Korea	Infector
1	Imported	F	China	Jan 18	Jan 20	Feb 6	-	Jan 19	-
2	Imported	M	China	Jan 10	Jan 24	Feb 5	-	Jan 22	-
3	Imported	M	China	Jan 22	Jan 26	Feb 12	-	Jan 20	-
4	Imported	M	China	Jan 21	Jan 27	Feb 9	-	Jan 20	-
5	Imported	M	China	Jan 26	Jan 30	-	-	Jan 24	-
6	Primary	M	-	Jan 26	Jan 30	Feb 19	Jan 22	-	3
7	Imported	M	China	Jan 26	Jan 30	Feb 15	-	Jan 23	-
8	Imported	M	China	Jan 21	Jan 31	Feb 12	-	Jan 23	-
9	Primary	F	None	Jan 30	Jan 31	Feb 24	Jan 26	-	5
10	Secondary	F	-	Jan 29	Jan 31	Feb 19	Jan 26	-	6
11	Secondary	M	-	Jan 30	Jan 31	Feb 10	Jan 26	-	6
12	Imported	M	Japan	Jan 20	Feb 1	Feb 18	-	Jan 19	-
13	Imported	M	China	Feb 1	Feb 2	Feb 24	-	Jan 31	-
14	Primary	F	-	Jan 29	Feb 2	Feb 18	Jan 20	-	12
15	Imported	M	China	Feb 1	Feb 2	Feb 24	-	Jan 20	-
16	Imported	F	Thailand	Jan 25	Feb 4	Feb 19	-	Jan 19	-
17	Imported	M	Singapore	Jan 24	Feb 5	Feb 12	-	Jan 24	-
18	Primary	F	Thailand	-	Feb 5	Feb 19	-	Jan 19	16
19	Imported	M	Singapore	Jan 31	Feb 5	Feb 21	-	Jan 23	-
20	Primary	F	-	Feb 4	Feb 5	Feb 24	Feb 1	-	15
21	Secondary	F	-	Jan 30	Feb 5	Feb 29	Jan 26	-	6
22	Primary	M	-	-	Feb 6	Feb 15	-	-	16
23	Imported	F	China	Feb 3	Feb 6	Feb 29	-	Jan 23	-
24	Imported	M	China	Feb 2	Feb 6	Feb 27	-	Jan 31	-
25	Primary	F	-	Feb 6	Feb 9	Feb 22	Feb 1	-	27
26	Primary	M	China	Feb 8	Feb 9	-	Jan 24	Jan 31	27
27	Imported	F	China	Jan 24	Feb 9	-	-	Jan 31	-
28	Primary	F	-	-	Feb 10	Feb 17	-	Jan 20	3

The information of 28 patients of COVID-19 between January 20 and February 17 was collected from the reports provided by the KDCA [1], the Korea Government Briefing Room [2] and the previous studies by Ki (Appendix 1 of [3]) and Hyun [4]. Note that no symptoms were observed for the patients (ID18, ID22, ID28).

.

Table S2. Definitions of probability distributions

Distribution	Probability density function	Mean	Variance
Birnbaum-Saunders	$f(x;\alpha,\beta)=\frac{\sqrt{\frac{x}{\beta}}-\sqrt{\frac{\beta}{x}}}{2\alpha x}\cdot\phi\left(\frac{\sqrt{\frac{x}{\beta}}-\sqrt{\frac{\beta}{x}}}{\alpha}\right)$	$\beta\left(1+\frac{\alpha^2}{2}\right)$	$\alpha\beta^2\left(1+\frac{5\alpha^2}{4}\right)$
Inverse-Gaussian	$f(x;\mu,\lambda)=\sqrt{\frac{\lambda}{2\pi x^3}}\cdot exp\left(\frac{-\lambda(x-\mu)^2}{2\mu^2 x}\right)$	μ	$\frac{\mu^3}{\lambda}$
Nakagami	$f(x;\mu,\omega)=\left(\frac{\mu}{\omega}\right)^{\mu}\frac{2}{\Gamma(\mu)}x^{2\mu-1}\cdot exp\left(\frac{-\mu}{\omega}x^2\right)$	$\frac{\Gamma\left(\mu+\frac{1}{2}\right)}{\Gamma(\mu)}\cdot\sqrt{\frac{\omega}{\mu}}$	$\omega\left(1-\frac{1}{\mu}\left(\frac{\Gamma\left(\mu+\frac{1}{2}\right)}{\Gamma(\mu)}\right)^2\right)$

Table S3. Estimations of epidemiological periods P_1-P_6 using the seven probability distributions

Distribution	Symptom onset to confirmation of imported cases (P_1)				Symptom onset to confirmation of local cases (P_2)				Confirmation to discharge (P_3)			
	Mean	SD	AICc	BIC	Mean	SD	AICc	BIC	Mean	SD	AICc	BIC
Exponential	6.75	6.75	95.39	95.88	2.56	2.56	37.46	37.09	15.88	15.88	182.89	183.89
Gamma	6.75	5.02	95.22	95.84	2.56	1.68	38.01	36.41	15.88	5.13	149.42	151.21
Weibull	6.76	4.74	95.11	95.73	2.57	1.66	38.34	36.73	15.91	4.66	147.10	148.88
Lognormal	7.13	7.28	96.38	97.01	2.57	1.99	37.51	35.90	15.96	5.61	151.25	153.03
Birnbaum-Saunders	6.69	6.10	95.84	96.46	2.56	1.84	36.97	35.36	15.87	5.56	151.22	153.01
Inverse-Gaussian	6.75	6.69	96.68	97.30	2.56	1.92	36.97	35.37	15.88	5.58	151.35	153.13
Nakagami	6.81	4.60	95.07	95.69	2.61	1.62	38.51	36.91	15.85	4.88	148.21	149.99
**Distribution**	**Symptom onset to discharge (P_4)**				**Incubation period (P_5)**				**Serial interval (P_6)**			
	Mean	SD	AICc	BIC	Mean	SD	AICc	BIC	Mean	SD	AICc	BIC
Exponential	21.90	21.90	173.85	174.69	5.67	5.67	51.79	51.42	6.56	6.56	54.42	54.04
Gamma	21.90	4.44	126.26	127.68	5.67	3.04	49.69	48.08	6.56	3.93	53.81	52.20
Weibull	21.87	4.29	124.30	125.72	5.73	3.46	51.39	49.78	6.63	4.13	54.70	53.10
Lognormal	21.94	4.69	127.79	129.21	5.53	2.96	47.65	46.04	6.45	4.16	52.45	50.85
Birnbaum-Saunders	21.90	4.71	127.99	129.41	5.68	3.00	47.69	46.09	6.57	4.06	52.16	50.55
Inverse-Gaussian	21.90	4.71	128.03	129.45	5.67	3.04	47.47	45.87	6.56	4.14	52.00	50.39
Nakagami	21.89	4.28	125.21	126.64	5.92	3.29	51.80	50.19	6.78	4.00	55.02	53.42

Figure S1. Fitted distributions for epidemiological periods P_1-P_6

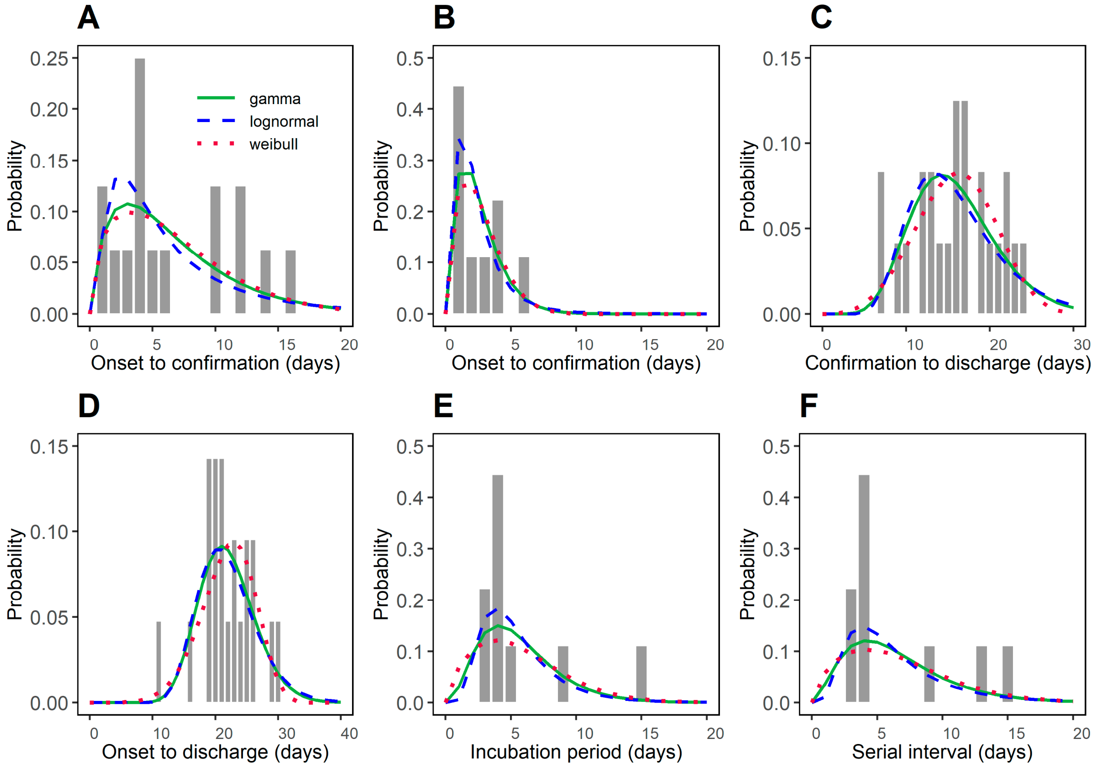

Fitted distributions for epidemiological periods P_1–P_6 using three probability distributions. Bars represent the observed data. (**A**) Reporting delay of imported cases (P_1: symptom onset to confirmation). (**B**) Reporting delay for local cases (P_2: symptom onset to confirmation). (**C**) Time between the confirmation and the discharge from the hospital (P_3: confirmation to discharge). (**D**) Time between the symptom on-set and the discharge from the hospital (P_4: symptom onset to discharge). (**E**) Incubation period (P_5). (**F**) Serial interval (P_6).

Figure S2. Fitted distributions for the reporting delay of imported cases between the entry to confirmation.

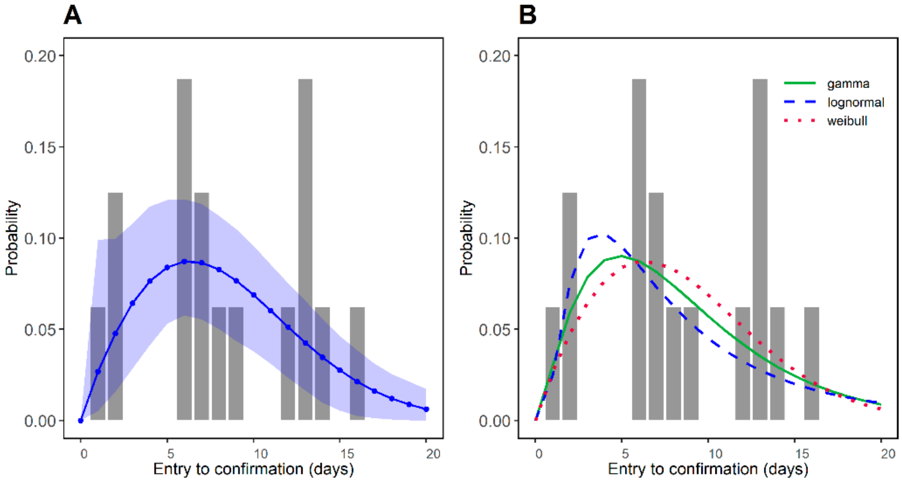

Fitted distributions for the reporting delay of imported cases between the entry to confirmation, using three probability distributions (gamma, log-normal, Weibull). Bars represent the observed data. (**A**) Blue dots represent the estimated values from the best-fitted Weibull distribution. Blue shaded region represents the 95% CI from 1000 samples. (**B**) Comparison of the fitted distributions for the reporting delay of imported cases between the entry to confirmation.

References

1. Korea Disease Control and Prevention Agency (KDCA). Press release. Available online: https://www.cdc.go.kr/board/board.es?mid=a20501000000&bid=0015 (accessed on 30 January 2021).
2. Korea Government Briefing Room. Press release. Available online: https://www.korea.net/Government/Briefing-Room/Press-Releases (accessed on 30 January 2021).
3. Ki, M., Epidemiologic characteristics of early cases with 2019 novel coronavirus (2019-nCoV) disease in Korea. *Epidemiology and health*, **2020**, 42, e2020007.
4. Hyun J.; Lee J.H.; Park Y.; Jung E.K., Interim Epidemiological and clinical characteristic of COVID-19 28 cases in South Korea, *Public health weekly report*, KCDA, **2020**, 13 (9).
